# 7.L. Workshop: New roles and innovative skill-mix in nursing across Europe: trends and lessons for implementation

**DOI:** 10.1093/eurpub/ckac129.446

**Published:** 2022-10-25

**Authors:** Organised by: WHO Europe, Technische Universität Berlin, Chair persons: Margrieta Langins (Canada), Claudia Maier (Germany)

## Abstract

Many countries in Europe are changing the roles and skill-mix of their nursing workforce. A sufficient and well-skilled nursing workforce has shown to be key for population health, most recently demonstrated during the Covid-19 pandemic. An increasing number of countries have introduced Master-level education for nurses to work in advanced practice nursing (APN) roles. These changes in the educational systems have implications on patients, clinical practice, teams and health systems. Moreover, many countries are re-orienting their health services from hospital to primary health care and prevention, driving new roles for nurses. The relevance of high-quality education and upskilling has been exemplified during the Covid-19 pandemic, where nurses had to quickly adapt to new treatment and caregiving situations in challenging work environments. Objectives of the workshop. This workshop will provide an overview of research and policy developments in Europe, drawing on recent research. First, an overview of innovative skill-mix changes in nursing will be presented and evidence on health outcomes, based on a study from the European Observatory on Health Systems and Policies. Second, research from Germany in five innovator hospitals will be presented, a country which is still at an early stage with integrating nurses in advanced roles. The panel discussion will address innovations in APN research, practice and policy lessons in Europe and with in-depth insights from four countries (Netherlands, Finland, Switzerland, Germany). In the Netherlands, the policy instrument “experimental law” has led to full practice authority for Nurse Practitioners, its impact on practice as well as barriers and enablers will be addressed. In Finland, the focus will be on recent evidence on the expanded role of nurse prescribers, the policy process and early evidence on impacts for nurses and patients. In Switzerland, the lack of regulation and reimbursement schemes restrict advanced practice, whereas drivers comprise changes in population needs, education (master and doctoral level) and reduced workforce availability. Developments at the national and cantonal level and the evaluation of innovative care models with APNs will be presented using case examples and recent research (e.g. university hospitals, primary care). The panelists will discuss implications for research, policy and nursing management, also taking into account the Covid-19 pandemic and other unexpected “health shocks” which have shown to impact considerably on nurses’ roles in practice. Lessons will be shared on strategies for a well-qualified and -resourced nursing workforce integrated in systems. The format is ‘regular workshop’ with two presentations and a panel discussion with experts from four countries.

**Key messages:**

• Multiple innovative nursing skill-mix model exist, with promising outcomes for individuals and population groups if nurses are well trained and equipped.

• Implementation of APN roles requires enabling policy contexts, sufficient funding, clinical leadership, teams that are open to innovations, and strong nurses that take on APN roles.

